# The role of metacognitive experience: Can feedback training affect kindergarteners'monitoring accuracy?

**DOI:** 10.1007/s11409-025-09422-4

**Published:** 2025-06-05

**Authors:** Kristin Kolloff, Ebru Ger, Claudia M. Roebers

**Affiliations:** Department of Developmental Psychology, Institute of Psychology, University of Bern, Bern, Switzerland

**Keywords:** Metacognition, Monitoring, Monitoring accuracy, Feedback, Cue utilization, Training, Children

## Abstract

This study explored the effects of metacognitive training on 6-year-old kindergarteners, focusing on (a) enhancing metacognitive monitoring accuracy through feedback, and (b) examining whether children use choice latency as a valid cue for their monitoring judgments (cue utilization). A total of 214 children were assigned to one of three groups: performance feedback, monitoring feedback, and an active control. They completed 12 training sessions and were tested before and after the training with memory learning tasks. Results showed that neither feedback type improved monitoring accuracy. However, choice latency was confirmed as a valid cue that children use in their confidence judgments. Despite repeated feedback, there was no observable integration of this feedback into future monitoring judgments. The study discusses both the theoretical and practical implications of these findings, highlighting the children’s cue utilization and suggesting avenues for future research.

**Supplementary Information:**

The online version contains supplementary material available at https://doi.org/10.1007/s11409-025-09422-4.

Imagine Alex, a 6-year-old boy who was excited to help his mother make her famous chocolate chip cookies. She asked him to get the sugar from a shelf in the kitchen. Even though he did not know which jar contained sugar, Alex decided to pour one into the mixing bowl. However, it was salt! The mistake meant the cookies did not taste right. But Alex learned something important – realizing that he is not sure is crucial – as he might consider things to reduce uncertainty, for example, asking for help. As the example illustrates, detecting uncertainty is ubiquitous in children’s and adults’ daily lives, and not surprisingly, metacognitive monitoring plays a dominant role in self-regulated learning across various academic domains (Dunlosky & Metcalfe, 2009). The present study’s starting point is young children’s emerging monitoring skills. In an attempt to steer this development in the direction of accurate monitoring as early as possible, we provide multiple occasions for monitoring and give feedback on either performance or monitoring accuracy and systematically address the potential benefits of such an intervention.

Metacognitive monitoring, defined as evaluating one’s learning (Nelson & Narens, 1990), is crucial for self-regulatory processes like strategy selection (Dunlosky & Rawson, 2012), study time allocation or termination of learning (van Loon & Oeri, 2023), and help-seeking (Coughlin et al., 2015). Actively engaging in monitoring, which involves assessing and reflecting on one’s learning and thinking processes, helps individuals to identify and correct potential errors. Consequently, individuals who can effectively monitor their learning and recognize their mistakes tend to perform better (Roebers et al., 2014).

Children begin developing monitoring skills as early as kindergarten and continue refining them throughout early elementary school (Schneider & Löffler, 2016; van Loon & Roebers, 2021). At this early age and important for the present study, children are already able to metacognitively distinguish between correct and incorrect answers metacognitively, typically showing greater confidence in correct than in incorrect responses (Destan & Roebers, 2015; Roebers, 2017; Schneider & Löffler, 2016; van Loon & Roebers, 2021). Nevertheless, kindergarten and primary school children’s over-optimistic view of their performance, especially when they err, strongly impedes their monitoring accuracy. Although there is a consensus in the literature that high confidence can have positive effects on children’s educational efforts and motivation, encouraging them to embrace and persist through new challenges (Bjorklund & Bering, 2002; Bjorklund & Green, 1992; Usher, 2009; Usher & Pajares, 2008), there are also serious drawbacks to excessive confidence. Overconfidence can impede learning because the ability to identify and correct one’s errors relies partly on accurately tracking one’s performance (Dunlosky & Rawson, 2012; Hartwig & Dunlosky, 2014). In other words, optimism about their abilities can lead children to set ambitious goals, but the balance between accurate metacognitive monitoring and confidence is crucial for their cognitive development and long-term academic success (Shin et al., 2007). In addition, if individuals do not learn to monitor their learning early on, poor monitoring and learning habits can become ingrained and continue to hinder educational success (Blair & Raver, 2015; Dunlosky & Metcalfe, 2009). Early intervention may thus be a key to improving monitoring in a sustainable way. There is a current debate as to whether children (a) simply disregard their performance allowing them to stay confident or (b) are reluctant to report on their uncertainty (Buehler et al., 2025; Kolloff et al., 2023).

Given the need to help children develop accurate monitoring abilities already from an early age, it is notable that only very few interventions target these skills in young children. Our study presents a novel metacognitive monitoring intervention with feedback for kindergarten-aged children. We realized a large-scale study and applied a memory paradigm designed to offer children diverse metacognitive experiences and a higher level of challenge, incorporating multiple cues to inform monitoring judgments—most notably, a wide range of item retrieval fluency, recognized as a highly valid and effective cue (Serra & Metcalfe, 2009). To find out whether young children disregard their performance or are reluctant to report on their uncertainty, our metacognitive training incorporates two different feedback forms, allowing us to investigate whether and how effectively children integrate these experiences with the feedback into their monitoring judgments. Additionally, our study expands on previous research on cue utilization by investigating whether kindergarten children can learn to use response speed (i.e., retrieval fluency, operationalized as choice latency in a memory recognition test) to inform their metacognitive monitoring. We explore whether and to what extent young learners can improve their reliance on choice latency as a cue for their monitoring judgments in recognition tests. Our intervention approach stands out from previous intervention efforts by including a large sample, an intensive training period (12 sessions), a computerized method that is modern, economical in terms of human resources, and allows upscaling (via Google Play Store®, for example), and the use of response times to explore cue utilization, an important aspect that has been neglected in previous studies (see Dignath et al., 2023 for a review; van Loon & Roebers, 2021).

Metacognitive monitoring accuracy is typically assessed by asking participants to rate their confidence either before (e.g., judgments-of-learning [JOLs]) or after studying (e.g., confidence judgments [CJs]; Dunlosky & Metcalfe, 2009; Nelson & Narens, 1990). With CJs, a retrospective measure, researchers ask participants to rate how sure they are that they recalled the information correctly. These judgments are typically made on item-specific Likert scales with a continuum ranging from “very sure” to “very unsure.” When assessing metacognitive monitoring in this way, two key measures are distinguished: relative and absolute monitoring accuracy. *Relative monitoring accuracy* reflects participants’ ability to discriminate between correct and incorrect answers based on their confidence ratings (discrimination/sensitivity). In contrast, *absolute monitoring accuracy* measures (bias) how well participants’ confidence levels align with their actual performance (for a review of different metacognitive monitoring measures, see Schraw, 2009). In the present study, we use CJs to examine both relative and absolute monitoring accuracy (our primary outcome variables). Relative accuracy was measured through item-specific confidence judgments made after a recognition test, reflecting participants’ ability to differentiate between correct and incorrect answers (monitoring discrimination). Absolute monitoring accuracy was assessed by comparing participants’ confidence judgments with their actual performance, determining how well they could estimate their recognition performance (bias).

## Metacognitive experience

The current study integrates two theoretical models to underpin the intervention approach. Efklides’ multifaceted and multilevel model of metacognition (2008) provides a broad developmental and educational perspective, framing the role of metacognitive experiences for metacognitive monitoring, while Koriat’s cue utilization framework (1997) focuses on the micro-processes occurring during monitoring task mastery.

Efklides (2008) emphasizes the importance of metacognitive experiences as a critical aspect for fostering and developing accurate monitoring skills. Metacognitive experiences are understood as the underlying and overt emotions and motivations that accompany both the preparedness to learn and learning (e.g., joy of success, feeling of mastery, motivation for accuracy). These experiences, in turn, shape the progress of a task by triggering processes of monitoring, control, and strategy use. For example, when children are repeatedly confronted with uncertain situations or outcomes, their metacognitive monitoring skills are shaped by feelings of certainty and uncertainty, competence, invested effort, and difficulty (Ghetti et al., 2013). With repeated task experiences, metacognitive experiences are refined through a calibration process, where monitoring processes should slowly become more accurate and aligned with performance. Further, metacognitive monitoring judgments are also shaped by social interactions and feedback from others, such as teachers, parents, and peers (Efklides, 2006, 2008, 2011). This teacher feedback is assumed to rank among the most influential factors in preparing children to handle school tasks, engage actively in learning, stay motivated, and achieve long-term academic success (Pozuelos et al., 2019). As Hattie and Timperley (2007) have outlined, feedback should be immediate and include positive reinforcement to maintain motivation, especially for younger children. Furthermore, children should receive developmentally appropriate feedback that is simple and concise, focuses on specific aspects of performance, and clearly differentiates between correct and incorrect responses (Hattie & Timperley, 2007; Pashler et al., 2005). As children grow older, feedback can become more detailed, encouraging self-assessment and prompting reflection on their work (van Loon et al., 2017). Thus, both feedback on performance and feedback on the alignment of monitoring with performance can – theoretically – be powerful tools to improve young children’s metacognitive monitoring.

Koriat’s (1997) cue utilization approach specifically targets the micro-processes inherent in metacognitive experiences during learning and memory tasks. From this perspective, individuals come to discover mnemonic rules of thumb, known as heuristics, and use these as cues to inform their monitoring. Variations in retrieval fluency, ease of recall, or even familiarity serve as mnemonic cues for metacognitive monitoring (Serra & Metcalfe, 2009). For instance, when a student is asked to list elements from the periodic table in a quiz, those elements they studied recently or repeatedly might come to mind more readily and receive higher monitoring judgments, while less familiar ones might take longer to recall and – even when recalled correctly – receiver lower monitoring judgments.

The cue utilization framework emphasizes the critical role of mnemonic cues as powerful information sources in shaping metacognitive judgments and assessing memory performance effectively (de Bruin & van Merriënboer, 2017; van Loon et al., 2017). For example, in both adults and children, retrieval fluency typically functions as a valid mnemonic cue, and its use is consistently related to monitoring accuracy (Serra & Metcalfe, 2009). According to Efklides and Schwartz (2024), young children’s monitoring judgments tend to be inaccurate due to their ignorance and underdeveloped recognition and use of valid task-inherent cues. The degree to which children discover and use valid cues, however, increases with metacognitive experiences and – in the long run – helps to develop more accurate monitoring skills (Efklides, 2006). Several studies on cue utilization have shown that as children get older, they benefit from prior experiences by detecting and using cues for their monitoring judgments (Ackerman & Koriat, 2011; Geurten et al., 2017; Roebers et al., 2019; van Loon et al., 2017).

In memory and learning research, numerous studies with adults have documented that confidence judgments are often linked to the speed of information retrieval (Benjamin & Bjork, 1996; Benjamin et al., 1998; Schwartz, 2024). Specifically, faster retrieval or selection of an answer is typically associated with higher confidence, suggesting that fast response times are more likely to indicate a correct answer, whereas slow response times indicate an incorrect answer, demonstrating a negative correlation between recall time and confidence level (Kelley & Lindsay, 1993; Nelson & Narens, 1990; Zakay & Tuvia, 1998). Research examining the association between retrieval fluency and learners’ confidence judgments has primarily involved adults (Benjamin et al., 1998; Karpicke, 2009; Schwartz, 2024; Serra & Dunlosky, 2005). Roebers et al. (2019) investigated the developmental trajectory of cue utilization in confidence judgments among 8- and 10-year-olds, assessed three times over two school years. Their findings revealed that longer response times were consistently linked to lower confidence, while shorter response times were associated with higher confidence—aligning with earlier studies (Ackerman & Koriat, 2011; Koriat & Ackerman, 2010). Notably, 8-year-olds in Roebers et al.’s study showed significant improvement in cue utilization over the year, gradually showing a comparable cue use as 10-year-olds, whose cue use did not further increase. This improvement in younger students likely reflects their growing experiences with learning tasks and memory failures, helping them better recognize and use cues to improve their monitoring accuracy. Moreover, in this study, individual differences in cue utilization were predictive for subsequent memory performance, rendering cue use an important, often overlooked developmental factor within memory development. This represents a significant gap in our understanding of metacognitive monitoring abilities in young samples and the factors driving its development. Hence, we sought to include children’s cue utilization: the computerized training allowed using response times in the recognition test to explore to what extent children (a) used these varying response times to inform their monitoring and (b) whether this changed over the course of the intervention.

## Feedback

According to Efklides (2008), over and above the metacognitive experiences on the task level, the accuracy of children’s monitoring judgments can also be enhanced and adjusted at the social level. Feedback at the social level, whether provided by a teacher, peer, or computer, is an essential component in children’s metacognitive monitoring learning process as it allows children to identify gaps and provides learners with information about how well they are performing (Butler & Winne, 1995). Winne (1997) suggested that frequent practice with feedback helps children refine their monitoring abilities over time, as it can reveal errors and difficulties they may not have noticed previously (Stipek & Tannatt, 1984). Indeed, research shows that high-quality feedback provided to young children in early education settings can significantly contribute to their long-term success (Pakarinen et al., 2017). Feedback can take different forms, and its impact on metacognitive skills depends on how well it integrates into the learner’s monitoring processes (Hattie & Timperley, 2007; van Loon & Roebers, 2021). Given the limited availability of intervention research for young children, we chose to compare one of the most often used feedback forms in education—performance feedback—with a more specific form of feedback, monitoring feedback, targeting to what degree monitoring aligns with performance. Performance feedback provides children with objective information about the correctness of their responses but lacks specific guidance on adjusting monitoring (Labuhn et al., 2010). In contrast, monitoring feedback highlights discrepancies between confidence judgments and actual performance, allowing for greater self-regulation and refinement of metacognitive monitoring (Callender et al., 2016; Geurten et al., 2017; van Loon & Roebers, 2020).

The few existing studies investigating the effect of feedback on young children’s metacognitive skills have produced inconsistent results, likely due to differing methods and feedback types. Notably, only a handful of studies have combined feedback with a structured intervention to enhance metacognitive monitoring in young children. One such study, entirely unrelated to the current data, is a recent training study with 7-to-8-year-old primary school children, examining how different types of feedback impact monitoring accuracy within a memory learning task (Buehler et al., 2025). Children participated in six computerized sessions and were assigned to one of three groups: metacognitive feedback, performance feedback, or an active control group. Results indicated that only metacognitive feedback led to a significant improvement in monitoring accuracy at posttest, while performance feedback had no effect. Van Loon and Roebers (2020) took a more interactive approach by providing face-to-face feedback directly through an experimenter using a problem-solving, thus, not a memory paradigm. In their study, 6-year-old kindergarteners received either monitoring feedback, performance feedback, or no feedback on each task item immediately after they had provided a solution and reported on their confidence. The results showed that monitoring feedback was more effective than both performance feedback and no feedback in helping children recognize their mistakes. Nonetheless, around 70% of errors remained undetected despite the feedback; that is, children were highly confident even for their errors, highlighting significant potential for further improvement. In one other study, van Loon et al. (2017) studied memory monitoring in 5- to 6-year-olds and found that after receiving performance feedback on response accuracy, children were able to re-evaluate some of their answers, and confidence judgments were then given with somewhat greater accuracy. Thus, both forms of feedback might yield to improvements in young children’s monitoring.

However, there is also contrasting research showing that despite repeated practice across multiple trials, children often find it challenging to effectively use performance feedback (e.g., Lipko-Speed et al., 2014). In a study by Lipko et al. (2009), the ability of 4- to 5-year-olds to adjust their confidence over several trials of a memory task was evaluated. Results indicated that despite repeated feedback, children continued to exhibit overconfidence, suggesting they did not effectively incorporate the feedback into their metacognitive predictions. Similarly, O’Leary and Sloutsky (2017) aimed to determine how the presence of feedback influences children’s ability to monitor their performance across 30 test trials, with 5-year-olds providing confidence judgments. Although task monitoring was effectively enhanced by external feedback, performance monitoring remained unaffected. Children consistently overestimated their performance regardless of whether feedback was provided, and most were unable to accurately determine whether their performance was better in the easier or the more difficult task.

Together, these inconsistent findings across different studies suggest that the efficacy of feedback can vary greatly depending on the task complexity, the age of the children, and the specific type of feedback provided. Yet, this approach has rarely been tested in a structured, multi-session intervention. Our study is highly innovative and aims to bridge this gap by investigating the effects of both performance and monitoring feedback in a computer-based training program designed to enhance metacognitive monitoring in kindergarten-aged children. The poor existing evidence underscores the need for more detailed research to determine how feedback mechanisms can be optimized to support emerging metacognitive monitoring skills in young children. Our study, therefore, takes a novel approach by integrating extensive metacognitive experiences within a structured app-based, thus resource-sparring intervention, allowing for repeated exposure to both performance and monitoring feedback as early as possible in the course of metacognitive development. We expect that monitoring feedback will lead to greater improvements in monitoring accuracy by providing young children with explicit insights into the accuracy of their confidence judgments, thus aiming to prevent the persistence of overconfidence. Additionally, our study extends previous research by exploring whether kindergarteners can learn to use task-inherent cues, such as response latency, to inform their monitoring judgments. By integrating these elements into a structured intervention, we seek to provide new insights into how young children develop metacognitive monitoring skills and how feedback mechanisms can be optimized for early childhood education.

## The present study

Over the past two decades, there has been a notable rise in the development of computer-based programs for early childhood education, including tools for phonological awareness (e.g., PADP®; Parry et al., 2024), early numeracy (e.g., MATHGarden®; Otterborn et al., 2019), and executive functions (e.g., COGMED®; Aksayli et al., 2019). However, despite these advancements, research on whether and how digital learning technologies support metacognitive monitoring remains scarce. While some programs provide personalized feedback (Verhoeven et al., 2020), their impact on young children’s ability to assess and regulate their own learning has received little attention. Our study addresses this gap by implementing a computer-based task (learning app) specifically designed to enhance metacognitive monitoring in children. This format has, at least in our opinion, many advantages. For one, a digital format allows for precise and standardized measurement of response latencies, which is essential for investigating cue utilization and monitoring processes in this study. For another, digital tasks offer an engaging and intuitive interface, minimizing experimenter variability while ensuring that all participants receive identical instructions and feedback. Given that children today are increasingly accustomed to digital learning environments, this approach is modern and offers an efficient approach to introduce multiple metacognitive experiences to children, yet in a highly controlled setting. While computerized interventions may require some adult supervision, their structured nature helps mitigate this limitation. By leveraging resource-efficient online applications, this study pioneers a novel approach to fostering metacognitive monitoring in young learners in times of digitalization that has by now also reached kindergarten classrooms.

As mentioned above, the training involved computerized, self-paced, memory-based learning tasks in which children were exposed to successful and unsuccessful outcomes. Children in the metacognitive training group repeatedly gave metacognitive judgments during memory tasks, providing multiple experiences of task-inherent cues like retrieval fluency (Efklides, 2008, 2011; Koriat, 1997; Koriat & Levy-Sadot, 1999). Moreover, after each trial, they received feedback on monitoring and/or performance. We expected a decrease in overconfidence (*bias; absolute monitoring accuracy*) from pre- to posttest through both types of feedback compared to the active control group. Specifically, we expected the monitoring feedback to reduce overconfidence (absolute monitoring accuracy) more strongly than the performance feedback. Additionally, we predicted an increase in *monitoring discrimination* (relative monitoring accuracy, i.e., the ability to metacognitively discriminate between correct and incorrect responses in the monitoring judgments) from pre- to posttest through both feedback compared to the control group, with monitoring feedback leading to greater improvements in monitoring than performance feedback alone (van Loon & Roebers, 2020). As data on both of our primary outcome variables during each training session was available from the learning app, we also addressed these two aspects of monitoring accuracy over the course of the training. By these means, we aimed to explore whether and after how many training sessions a benefit of the training might emerge.

Additionally, we aimed to explore whether kindergarteners can detect and use cues and thus benefit from repeated metacognitive experiences regarding the information they use when monitoring. Drawing on the limited existing research in primary children (Geurten et al., 2021; Koriat & Ackerman, 2010; Roebers et al., 2019), we hypothesized that shorter choice latencies in the recognition test would correlate with higher CJs, indicative of cue utilization and that through the training children would increasingly use easily available cues to inform their monitoring.

## Method

### Participants

Our study’s total sample comprised 222 participants, recruited from 26 public kindergartens in the larger vicinity of a mid-sized university town in Switzerland. In Switzerland, formal education starts at age 4 with two years of kindergarten, which is part of the required 11 years of schooling. The curriculum is nationally standardized and taught by qualified teachers. Two children opted out, and we excluded the data of one participant due to insufficient language skills. We excluded five more participants because there were missing data in the memory task on the posttest. Thus, the final sample consisted of *N* = 214 participants (49% girls;* M*_age_ = 6.4 years; *SD* = 0.4, age range between 5.5 to 7.7 years). The children were from both urban and rural regions, predominantly belonging to lower- to upper-middle-class families. The participants’ families originated from six geographic regions. The majority (70%) had roots in Western Europe, followed by 10% from Asia, 7% from Eastern Europe, and 5% from the Middle East, while 0.5% had African origins. Additionally, for 7% of participants, the family’s geographic background was unspecified or unknown. As to language proficiency, 66% of participants were native speakers of the instructional language, while 27% were non-native speakers but demonstrated sufficient proficiency to engage in the study. Participants were randomly assigned to one of three training groups: metacognitive training with monitoring feedback (*n* = 71), metacognitive training with performance feedback (*n* = 71), or active control group without feedback (*n* = 72). Parents gave written consent, and children gave oral assent to participate. The Faculty’s Ethics Committee at our university approved the study (further information withheld for reviewing), and it was carried out in accordance with the declaration of Helsinki.

### Design

We employed a pretest-training-posttest design spanning a total period of eight weeks. This study included two metacognitive training groups and one active control group.

All tasks used for the pretest, training, and posttest were presented on a tablet computer (Samsung Galaxy Tab S4 and Samsung Galaxy Tab A7) with a touch screen (10.4″ and 10.5″). They were carefully designed as child-friendly learning apps for young children to interact with using their fingers. This app-based approach not only made the learning process enjoyable but also enhanced the educational aspect of the task by combining entertainment with repeated practice in monitoring. Anonymized data was automatically synchronized to a secure server upon completion. Using headphones, the participants worked on the tasks individually in small groups supervised by a trained experimenter.

During the pretest and posttest sessions, children were tested in small groups ranging from three to 14 participants, while during the training, groups consisted of no more than eight participants per group. Trained research assistants provided general instructions regarding the test material, supervised children throughout, and offered individual technical support whenever needed. All tests were conducted in a tranquil setting within their kindergarten. The pretest sessions were conducted on two separate days, with a delay of at least one day and a maximum of two days between them. The 12-session training started within two weeks following the pretest and was conducted two to three times per week. The posttest was conducted within one week of the final training session. Our decision to opt for 12 training sessions is driven by a review of research on training duration’s effect on cognitive functions. Although several studies (Dignath et al., 2008; Kolloff et al., 2023; Wang & Sperling, 2021) have found no consistent effect of intervention duration on the effectiveness of metacognitive skills training, extensive research in the domain of executive functions suggests that longer training periods, characterized by consistent session lengths and frequencies, tend to lead to improved outcomes. This divergence highlights the complexity of the relationship between intervention length and training effectiveness across different cognitive domains (Ericsson, 2006; Ericsson & Towne, 2010; Jaeggi et al., 2008). We selected 12 sessions to provide ample practice time necessary for significant enhancements while remaining manageable for kindergarten children (Diamond & Ling, 2016).

## Material and procedure

### Pretest–posttest procedure

On the first day of the pretest, small groups of children were introduced to a storybook. The book was designed to promote an intuitive understanding of monitoring judgments and to prepare the children using confidence scales. We included two distinct rating scales. For the pretest and posttest, the rules of the hot–cold game were explained, and children had to rate their confidence on a 7-point Likert scale adapted from Koriat and Shitzer-Reichert (2002). This thermometer scale consisted of seven segments color-coded from deep blue (“very cold,” i.e., very uncertain) to red (“very hot,” i.e., very certain). The other scale introduced in the book and used during the training was a 4-point smiley scale with child-friendly smiley faces ranging from very uncertain to very certain. By actively practicing the use of two different rating scales, we aimed to prevent children from habitually selecting the same options on the confidence scale and applying monitoring judgment to different tasks. In other words, by using a different scale in the pre- and posttest compared to the training sessions allows to address true changes in monitoring (near-transfer). Both scales are shown in Appendix Figures B1 and B2. During the storybook reading, participants were actively encouraged to practice using both scales, regardless of their experimental condition assignment, to further prepare them for the upcoming tasks.

We utilized a paired-associates learning task with a recognition test and CJs to assess children’s monitoring ability. The task has been proven effective as learning material for younger children in previous studies (Destan, et al., 2014; Destan & Roebers, 2015). Thirty-two items were randomly divided into two sets, each containing 16 Japanese characters (Kanjis). For the pretest, participants were randomly assigned to either set A or B, and for the posttest, they were given the other set. The materials and procedures underwent a pilot test with a different sample to ensure sufficient variability in item difficulty and to achieve comparable levels of task difficulty at both measurement points. Based on the findings from the pilot study, items with an item difficulty index ranging from 0.11 (difficult) to 0.78 (easy) were selected for each measurement point (Moosbrugger & Kelava, 2008). This selection criterion ensured balanced levels of difficulty across the sets, with both comprising 22% easy, 60% average, and 16% difficult items.

The task instructions were delivered orally through headphones and displayed on the screen simultaneously with the instructions being presented on the screen. Children completed a practice trial to familiarize themselves with the task and had the opportunity to practice using the tablets. The test trials started as soon as the participants had successfully completed the practice trial. During the test, the experimenter supervised the participants.

The paired-associates task was divided into four distinct phases. A schematic of the task procedure is provided in Appendix Figure C1. The entire task lasted approximately 15 min. *Learning Phase:* Participants learned 16 Japanese characters (Kanji) and their corresponding pictorial meanings. For each child, items were presented in random order, with the participants given five seconds to study each picture pair. *Delay phase*: Following the learning phase, a one-minute filler activity was introduced to prevent rehearsal strategies. During this cat-and-mouse game, participants used their fingers to interact with an animated cat on the screen to catch a mouse. *Recognition Phase:* Participants then completed a recognition test. For each trial, one Kanji appeared on the left side of the screen, accompanied by four pictures on the right. Among the four presented alternatives, one picture matched the meaning of the Kanji, while the other three were associated with different learned picture pairs. The three alternatives were randomly selected for each participant. The recognition test had no time limit. *Monitoring Phase:* Immediately following each test trial, participants provided CJs on the thermometer scale by responding to the question: *“How sure are you that you selected the correct answer?”.*

### Monitoring training

For our training, we employed two distinct tasks: a memory learning task for the experimental groups and an executive function task for the active control group. This was done to prevent motivational issues in control children performing tasks without any feedback, which might not challenge them as much as experimental tasks, potentially affecting engagement and result validity. To ensure high motivation for participation and account for the Hawthorne effect, we chose varying versions of a cognitive control task for the active control group. This is crucial as studies showed that effect sizes are significantly reduced when active controls are used for comparison (Diamond & Ling, 2016; Melby-Lervåg & Hulme, 2013).

In the metacognitive training, we used the same memory learning paradigm. In contrast to the Kanji task, the training task, called “Lea and Luis,” was integrated into a cover story. This story revolved around two children, Lea and Luis, who were similar in age to the participants. The story described how they helped two male and female zookeepers with their daily tasks. In 12 sessions, each centered on these characters feeding different animals of 12 different species in their respective habitats (e.g., forest, ocean, desert). This narrative offered participants a meaningful and enjoyable context as they actively selected the appropriate food for each species. The materials and procedures underwent a pilot test with a different sample to ensure sufficient variability in item difficulty (Buehler et al., 2025; Kolloff et al., 2023). To ensure a smooth training procedure, one to two trained research assistants handled the technical setup, provided general instructions, supervised participants, and helped with the tablets and headphones whenever needed. In the first session, four observational learning trials were implemented, featuring four different item pairs. During these trials, the two protagonists demonstrated how to monitor their recognition performance and correctly use the smiley scale. The participants were provided detailed explanations about each CJ level corresponding to a specific smiley face. This extended explanation aimed to facilitate learning through observation. Once the participants successfully completed a practice trial, the test began. During the first session, the children learned eight items. Starting from the second session, they were given 12 paired-associates to study. In session two, the use of the smiley scale and, again, its detailed explanations were reiterated. We deliberately used a different scale during training to prevent children from memorizing specific response mappings and to encourage genuine metacognitive reflection. This variation ensures that improvements in the posttest would reflect a true transfer of monitoring abilities rather than simply memorized response tendencies. Thus, all participants worked on the same topic in sessions one and two. From session three to session twelve, the order of the session topics was based on a Latin square design.

The training task was structured into five distinct phases. The entire task spanned approximately 15 min. In the *learning phase,* participants learned 12 paired associations (eight items at session one of an animal and its preferred food, four practice trials). Each item was presented in a random sequence, with participants given five seconds to study each picture pair. In the *delay phase*, we designed six unique, short, interactive activities to inhibit the use of memory strategies. Each activity, lasting one minute, resembled the cat-and-mouse game from the pretest. Each of these activities was employed twice over the course of the training. During the *recognition*, the screen displayed an image of an animal on the left side, along with four different feed pictures on the right. Out of these, one was the correct feed choice for the displayed animal, whereas the other three alternatives corresponded to feed types associated with different animals related to the same topic and were already known to the participants. This recognition test proceeded without any time limitations. In the *monitoring phase*, children were asked to provide CJs immediately after each recognition trial. They rated their certainty regarding the accuracy of their responses using a 4-point smiley scale. Following each CJ, *during the feedback phase*, participants received visual feedback to reinforce learning: a green tick indicated a correct response, while a red cross denoted an incorrect one. Additionally, they received auditory feedback, depending on the experimental condition to which they were assigned. In the *Performance Feedback* group, participants were simply informed about the accuracy of their responses. In the *Monitoring Feedback* group*:* This feedback included the accuracy of their responses, along with whether participants’ CJ matched their performance, resulting in eight types of feedback. A detailed description of the feedback provided for both experimental feedback groups can be found in Table A1 in Appendix A.

#### Active control group

The activities in the active control group were structured around a variation of the Hearts and Flowers task, a cognitive control task adapted from Davidson and colleagues (Davidson et al., 2006; Diamond et al., 2007), capturing inhibition and cognitive flexibility component of executive function.

To complete the Hearts and Flowers task, three blocks were presented in a fixed order: Hearts, Flowers, and Mixed block. In all blocks, either a heart or a flower appeared on the right or left side of the screen. In the congruent block, children followed the rule to press the button on the same side as the heart appeared. The incongruent block required children to remember an additional rule: when a flower appeared, they were to press the button on the opposite side, suppressing the tendency to respond on the side where the stimulus appeared. In the mixed block, congruent and incongruent trials were interleaved, further increasing the demands on the participants’ cognitive flexibility. To maintain children’s motivation during the training, the tasks were game-like and integrated into a narrative featuring two different animals in each session overcoming various challenges (e.g., birds hurrying home before a storm). Identical to the metacognitive training, the active control group completed 12 sessions of similar length but differed in that participants did not receive any feedback on their performance. The active control group did not participate in any memory tasks during the training sessions.

### Measures

In this study, we assessed monitoring accuracy using classical measures; that is, we chose *bias* and *discrimination*. Although both measures relate to the accuracy of metacognitive monitoring, they represent different aspects and use different measurement methods. Bias assesses how accurately an individual’s estimated performance matches their actual performance in *absolute* terms. Conversely, discrimination assesses how accurately an individual can metacognitively discriminate between correct and incorrect responses (Bol & Hacker, 2012), independent of the level of confidence, thus allowing for accurate monitoring in *relative* terms.

We calculated *bias* as a measure of *absolute accuracy* to examine the magnitude of the difference between one’s CJ and actual performance to determine whether an individual is over- or underconfident (Schraw, 2009). For each participant, we calculated a bias score by first coding their CJs on a scale representing predicted recognition: 1 as 0%, 2 as 17%, 3 as 33%, 4 as 50%, 5 as 67%, 6 as 83%, and 7 as 100%. Memory performance was coded as 0% for incorrect and 100% for correct answers. The bias score was then determined by calculating the mean difference between the estimated recall and actual memory performance. The bias score can vary from −1 to 1, with values close to zero indicating perfect accuracy. A negative score signifies underestimation, while a positive score reflects overconfidence (Dunlosky & Metcalfe, 2009; Kälin & Roebers, 2022; Schraw, 2009).

We calculated *monitoring discrimination* as a measure of *relative accuracy,* the degree to which a person’s judgments can predict the likelihood of a correct answer relative to an incorrect answer (Bol & Hacker, 2012; Nelson, 1996). Therefore, we subtracted the mean CJs for incorrectly answered items from the mean CJs for correctly answered items (Dunlosky & Thiede, 2013; Roebers, 2002). Discrimination scores could range from −6 (poor discrimination) to + 6 (perfect discrimination), with a score near zero being indicative of unsystematic or random monitoring judgments. A positive discrimination score indicates that higher CJs are associated with correct answers and lower CJs with incorrect answers. Conversely, a negative indicates that lower CJs are given for correct answers and higher CJs for incorrect answers. A value near zero indicates that the judgments are not systematically related to the correctness of the answer (Schraw, 2009).

Although not the central focus of our study, we have also implemented an exploratory approach to examine whether and to what extent children are able to use retrieval fluency as a mnemonic cue and whether feedback will impact children’s cue utilization. We investigated the specific contributions of retrieval fluency as a cue, operationalized as choice latency in a recognition test. *Choice latency* was measured by the time (in seconds) needed to select an answer in the recognition test. To determine the extent to which kindergarteners use cues to inform their monitoring judgments, we calculated the Gamma correlation between choice latency and confidence judgments as an index of *cue utilization* (Koriat, 1995; Koriat & Ackerman, 2010).

However, despite reported developmental trends in cue utilization (Roebers et al., 2019), choice latency as a cue is only useful when it is a valid cue. In adults, choice latency has been found to be diagnostic of accuracy. That is, faster choice latencies are associated with a higher likelihood of the answer being correct compared to longer choice latencies, which are typically associated with a higher likelihood that the answer is incorrect. In accordance with previous research (Roebers et al., 2019), we thus assessed *cue validity* by computing a within-person Gamma correlation between choice latency and recognition correctness.

Memory performance was operationalized as percentage of correctly recognized items.

### Data analysis

The data in this study were analyzed using R statistical software, version 4.2.3 (R Core Team, 2022). Given the nested structure of the data, a multilevel model approach (MLM) was used to account for its dependency (e.g., Bliese et al., 2018; Chen & Chen, 2021; Finch et al., 2014; Hox et al., 2017). In our study, we implemented a two-level within-subject design. This involved within-students effects (repeated measures) at Level 1, nested in between-students effects (condition) at Level 2. For the analysis, we conducted a series of linear mixed-effects models (LMMs), progressing from intercept-only models to those including cross-level interaction effects. We use the *lmer* function and generalized linear mixed-effects model (GLMM) using the *glmer* function from the *lmeTest* package, version 1.1.32 (Bates et al., 2015). For the models, we employed Maximum Likelihood (ML) estimation. To compare model fit, we used the likelihood ratio test and the fit indices Akaike information criterion (AIC) and the Bayesian information criterion (BIC; Akaike, 1974; Raftery, 1995). For reasons of parsimony, we report only the final models that demonstrated the best fit. In determining the sample size for our study, we aligned our approach with those used in other training studies (de Bruin et al., 2017; Röthlisberger et al., 2012; Wang & Sperling, 2021).

## Results

In this section, we first present the analyses for pretests-posttests data. In the second part, we present the results of the training data collected over the course of the 12 sessions.

### Pretest and posttest data

In preparing the data for analysis in the pre- and posttest task (Kanji task), choice latencies below 500 ms were removed (2 of 6,848 observations, 0.03%), and outliers, defined as choice latencies >  ± 3 SD from the individual’s mean (71 of 6,846 observations, 1.04%) and group level mean (117 of 6,775 observations, 1.73%), were also excluded. Despite some missing data points, MLM use available data points to estimate model parameters, thus allowing for the inclusion of all participants in the analysis (Hox et al., 2017).

### Preliminary analysis

Table 1 offers the descriptive statistics for memory performance, CJs overall, and correct and incorrect recognition, respectively, as well as measures for absolute and relative monitoring accuracy (bias and discrimination) as a function of group and measurement point. Recognition accuracy ranged from 35 to 41%, independent of group, with an increase from pre- to posttest. To ensure that the groups did not differ from each other on the dependent variables before training, we used one-way ANOVAs. No significant group differences were found for bias, *F*(2, 211) = 0.25, *p* = 0.78, and discrimination, *F*(2, 211) = 0.75, *p* = 0.47, respectively.

**Table 1:** Mean CJs, recognition performance, bias, and discrimination as a function of measurement point and group

Group	Performance (%)	Mean CJs correct	Mean CJs incorrect	Bias	Discrimination
MC Monitoring Feedback					
Pretest	0.36 (0.20)	5.42 (1.68)	4.99 (1.50)	0.35 (0.54)	0.43 (1.26)
Posttest	0.41 (0.17)	5.55 (1.53)	4.92 (1.61)	0.29 (0.58)	0.63 (1.30)
MC Performance Feedback					
Pretest	0.35 (0.15)	5.38 (1.67)	4.73 (1.94)	0.32 (0.59)	0.65 (1.12)
Posttest	0.39 (0.18)	5.03 (1.96)	4.62 (2.07)	0.24 (0.61)	0.41 (1.22)
Active Control					
Pretest	0.35 (0.14)	5.45 (1.83)	5.00 (1.85)	0.35 (0.59)	0.45 (1.15)
Posttest	0.38 (0.17)	5.44 (1.61)	4.90 (1.83)	0.31 (0.59)	0.55 (1.05)
Overall					
Pretest	0.35 (0.17)	5.42 (1.72)	4.91 (1.77)	0.34 (0.58)	0.51 (1.18)
Posttest	0.40 (0.17)	5.34 (1.72)	4.82 (1.84)	0.28 (0.60)	0.53 (1.19)

*SD* in parentheses, CJs = Confidence judgments

#### The role of metacognitive experience and feedback on measures of monitoring accuracy

We investigated whether and how our two feedback groups influence children’s monitoring accuracy, focusing separately on two dependent variables: bias and discrimination. We used the following main predictors across both analyses: measurement point (pretest, posttest) at Level 1 and condition (MC Monitoring Feedback, MC Performance Feedback, AC) at Level 2. Participants were accounted for as random effects to control for variability due to repeated observations per participant. We defined the AC group as the intercept (i.e., reference group) and added fixed effects for the MT Monitoring Feedback and MT Performance Feedback group.

To test our hypotheses, we conducted two sets of analyses using a cross-level interaction model between measurement point and condition, one for bias and another for discrimination. We compared these models to more parsimonious models without the interaction terms. The analyses showed no significant improvement in model fit with the interaction term, neither for *bias*, χ^2^(2) = 0.39, *p* = 0.83, nor for *discrimination*, χ^2^(2) = 3.26, *p* = 0.20, indicating no differential training effects on both outcome variables. Consequently, for reasons of model parsimony, we decided to continue our analysis using conditional models without the interaction terms using the following syntax for bias: bias ~ measurementPoint + condition + (1 | subject), and for discrimination: discrimination ~ measurementPoint + condition + (1 | subject).

#### Absolute monitoring accuracy for pre- and posttest

The results for the conditional model for the *absolute monitoring accuracy* revealed that measurement point was a significant predictor (γ = −0.06, SE = 0.03, *t*(214) = −2.18, *p* = 0.03). We found no main effects for MC Monitoring Feedback (γ = −0.01, SE = 0.04, *t*(214) = −0.28, *p* = 0.78) and for MC Performance Feedback (γ = −0.05, SE = 0.03, *t*(214) = −1.22, *p* = 0.22). This result indicates that children reduced their bias scores, showing less overconfidence at posttest across the three groups, without significant variation in slopes between groups, partially confirming our hypothesis. Please refer to the Supplementary Material Table 1 for a detailed report of all models.

#### Relative monitoring accuracy for pre- and posttest

The results for the conditional model for the *relative monitoring accuracy* revealed that measurement point was not a significant predictor (γ = 0.02, SE = 0.10, *t*(214) = 0.19, *p* = 0.85). Further, we found no main effects for MC Monitoring Feedback yielded (γ = 0.03, SE = 0.15, *t*(214) = 2.13, *p* = 0.83) or for MC Performance Feedback (γ = −0.03, SE = 0.15, *t*(214) = −0.21, *p* = 0.83). Contrary to our expectations, overall – in contrast to overconfidence—children’s ability to discriminate between correct and incorrect answers did not change over the course of the training, independent of group. Please refer to the Supplementary Material Table 2 for a detailed model report.

**Table 2:** Mean recognition accuracy and choice latency for recognition as a function of measurement point and group

Group	CL correct (s)	CL incorrect (s)	Cue utilization	Cue validity
MC Monitoring Feedback				
Pretest	6.15 (1.70)	6.32 (1.94)	−0.21 (0.38)	−0.09 (0.39)
Posttest	4.98 (1.65)	4.95(2.15)	−0.13 (0.40)	−0.06 (0.35)
MC Performance Feedback				
Pretest	5.89 (1.62)	6.13 (1.94)	−0.09 (0.40)	−0.04 (0.36)
Posttest	4.96 (1.53)	4.75 (2.29)	−0.19 (0.38)	0.00 (0.39)
Active Control Group				
Pretest	5.84 (1.71)	6.09(1.85)	−0.05 (0.41)	−0.02 (0.36)
Posttest	5.51(1.46)	5.69 (2.04)	−0.19 (0.31)	−0.09 (0.36)
Overall				
Pretest	5.99 (1.68)	6.18 (1.91)	−0.12 (0.40)	−0.05 (0.37)
Posttest	5.15 (1.57)	5.13 (2.20)	−0.17 (0.37)	−0.05 (0.37)

*SD* in parentheses, s = seconds, CL = choice latency in recognition test; cue utilization = Gamma correlations between CJs and choice latencies in the recognition test (the shorter choice latencies, the higher the CJs); cue-validity = Gamma correlation between choice latencies in the recognition test and recognition correctness (the shorter choice latencies, the higher likelihood being correct)

#### The role of metacognitive experience and feedback on cue utilization

Table 2 presents the descriptive statistics for the choice latencies in the recognition test overall, and for correct and incorrect answers, as well as Gamma correlations for (a) the confidence-accuracy relation (cue validity) and (b) the confidence-choice latency relation (cue utilization), as a function of group and measurement point.

#### Cue validity

The mean choice latencies in the recognition test indicated that children across groups responded faster when giving correct answers than incorrect answers, both at the pretest and the posttest; that is, retrieval fluency appeared to be available for children. Importantly, the overall Gamma correlations between confidence judgments and recognition accuracy (i.e., cue validity) between choice latencies for recognition and confidence (i.e., cue utilization) were reliably different from zero (p < 0.001). To explore whether kindergarteners’ choice latencies differed significantly as a function of the correctness of their answer in the recognition test, we briefly examined whether correct recognition was associated with shorter choice latencies in recognition than incorrect recognition. Results revealed that retrieval fluency as a cue was available for the children, both pretest and posttest (γ = 0.18, SE = 0.07, *OR* = 1.20, z = 2.76, *p* < 0.01; see Appendix Figure D1). However, we found no significant training effects on cue validity as the three groups did not differ from each other at posttest in cue validity. Please refer to Supplementary Material Table 3 for the complete model report.

#### Cue utilization

Inspection of Table 2 reveals that the mean choice latencies of both MC training groups become shorter, both for correct and incorrect answers, in the posttest compared to the AC group. That is, it appears that children in the two MC training groups responded overall faster at the posttest compared to the active control group.

As the cue validity was indeed confirmed to be present in our young sample, we further investigated the impact of feedback on children’s *cue utilization*. To explore whether and to what extent kindergarteners used the time needed to select an answer in the recognition test to inform their monitoring judgments and whether this differs between groups, LMM models were run. Confidence judgments served as the dependent variable. Measurement points (pretest, posttest) and choice latencies in recognition (measured at the item level) served as main predictors at Level 1. Group (MC Monitoring Feedback, MC Performance Feedback, Active Control) was incorporated as a Level 2 predictor. Mean recognition accuracy per participant was included as a covariate to receive the net effect of monitoring judgments and proved to be significant (see Supplementary Material Table 4). In the model, we accounted for individual differences by treating participants as random effects. Specifically, we allowed both the intercept and the effect of time to vary across participants. First, we compared a conditional model that included measurement point, choice latencies in recognition, and condition as main predictors with a cross-level interaction model that incorporated an interaction term combining measurement point, choice latency, and group. We defined the AC group as the intercept (i.e., reference group). The cross-level interaction model did not provide a better fit, χ^2^(7) = 0.00, *p* = 1. Consequently, we ran the conditional model by using the following syntax: CJ ~ measurement point + choice latency + condition + recognition accuracy + (measurement point | subject). We included measurement point and participants as random effects in each model.

As expected, shorter choice latencies in the recognition test were significantly associated with higher CJs (γ = −0.06, SE = 0.01, *t*(6,543) = −7.93, *p* < 0.001), indicating substantial cue utilization. We found no main effect for MC Monitoring (γ = 0.02, SE = 0.22, *t*(214) = 0.10, *p* = 0.92) and MC Performance Feedback (γ = −0.28, SE = 0.22, *t*(214) = −1.29, *p* = 0.20) and measurement point (γ = −0.15, SE = 0.13, *t*(215) = −1.09, *p* = 0.28). Regardless of the measurement point, the results indicated that kindergarteners indeed considered choice latency as a valid cue when giving confidence judgments, with shorter choice latencies related to higher confidence judgments. However, training with either form of feedback did not impact their cue utilization. Find a detailed report of all models in the Supplementary Material Table 4.

#### Additional explorative analyses: Choice latencies as a function of confidence level

As indicated previously, even young children utilized choice latencies as valid cues to inform their CJs. This is evidenced by the observation that low CJs were associated with longer times taken to select an answer (the individual hesitates to give an answer), while high confidence correlates with shorter choice latencies. However, there is also research showing that when an individual is very unsure about an answer, CJs can be low and be given very quickly (knowing right away that she is unsure about the answer), resulting in an inverted U-shaped curve of choice latencies of confidence judgments as a function of level of confidence on the scale (e.g., van Loon et al., 2013). Thus, we aimed to explore whether feedback in connection with intensive feedback training can help children to more quickly differentiate between what they know and what they do not know. Similar to the findings of van Loon and colleagues (2017) and others (Benjamin et al., 1998; Koriat and Ma’ayan, 2005; Son & Metcalfe, 2005), this might enable them to make very low and very high confidence judgments more rapidly. As a result, one might expect inverted U-shaped curves when addressing the link between choice latencies and CJs levels, rather than a linear relationship as the Gamma correlations assess, with ambivalent or ambiguous item responses in the middle of the confidence scale yielding to the longest choice latencies (Nelson & Narens, 1990).

**Fig. 1:**
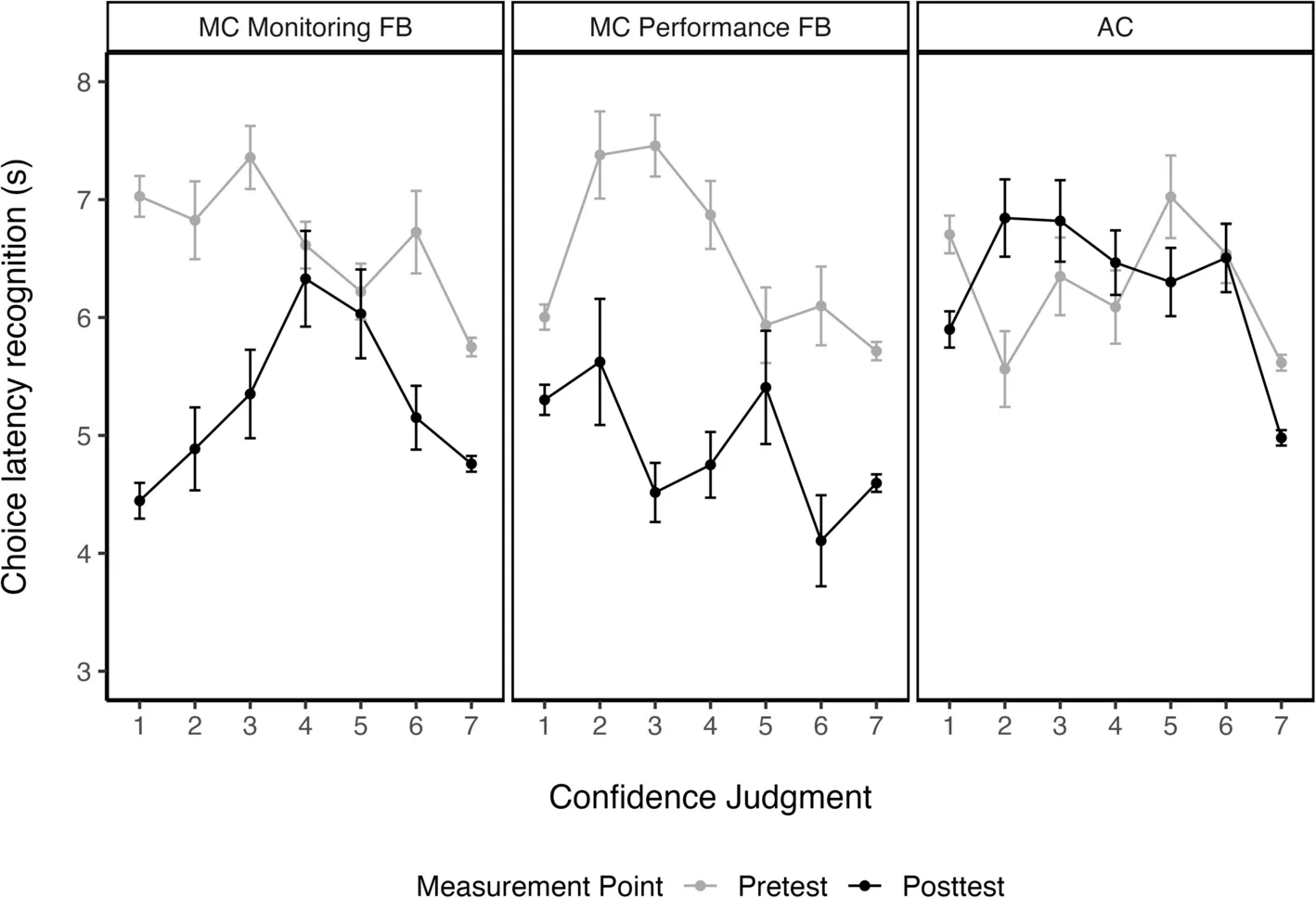
Choice latencies as function of group, measurement point, and confidence judgement level*.* Note. Error bars indicate standard errors

Figure 1 illustrates the choice latencies as a function of group and confidence levels. While the pretest data revealed the typical pattern of a linear and negative confidence-choice latency relationship across all groups, differences between groups became apparent in the posttest. Interestingly, only children in the MC Monitoring Feedback group significantly reduced reaction times for both *low and high* confidence levels. The trend in this group suggested that the relationship is evolving from a linear to a quadratic association, that is, an inverted U-shaped dynamic. Conversely, the MC Performance Feedback group displays an opposite pattern, where a relatively weak quadratic relationship shifts to a more linear relationship from pretest to posttest. The children in the AC group appeared not to change their pattern of choice latencies as a function of confidence level from pre- to posttest at all.

To test for a possible quadratic effect, we split the data by group and ran cross-level interaction models separately for each group. We included measurement point, CJ, and CJ as a quadratic term as the main predictors. Participants were included as random effects in each model. Choice latencies in recognition served as the dependent variable. We used the following syntax: choice latency ~ CJ + measurement point + I(CJ^2) + measurement point * CJ + measurement point * I(CJ^2) + (1 | subject).

For the MC Monitoring Feedback group, a quadratic cross-level-interaction model had the best fit, χ^2^(2) = 16.15, *p* < 0.001. The results also showed a significant linear interaction term (γ = 1.16, SE = 0.34, *t*(2,168) = 3.46, *p* < 0.001). The positive linear term indicated that at posttest, higher CJs were associated with shorter choice latencies. The negative significant quadratic interaction term suggested a shift towards an inverted U-shaped relationship at posttest, where very high and very low CJs were associated with *shorter* choice latencies (γ = −0.12, SE = 0.04, *t*(2,168) = −3.05, *p* < 0.01).

For the MC Performance Feedback group, too, the quadratic interaction model had the best fit, χ^2^(2) = 11.91, *p* = 0.01. The results also revealed a significant linear interaction (γ = −1.12, SE = 0.32, *t*(2,112) = −3.45, *p* < 0.01). As in the MC Monitoring FB group, higher CJs were associated with shorter choice latencies at posttest. The positive significant quadratic interaction term suggested a shift towards a slight U-shaped relationship at posttest, where very high and very low CJs were associated with *longer* choice latencies (γ = 0.13, SE = 0.04, *t*(2,112) = −3.44, *p* < 0.001).

For the AC group, we tested a quadratic conditional model against a quadratic interaction model, finding that the conditional model without the interaction term provided the best fit, χ^2^(2) = 3.97, *p* = 0.13. We found a significant main effect for the quadratic term of CJ (γ = −0.06, SE = 0.02, *t*(2,222) = −3.05, *p* < 0.01), indicating a slight tendency for an inverted U-shaped relationship. That is, very high and very low CJs were associated with shorter choice latencies. However, the effect was much less pronounced than in the MC Monitoring Feedback group.

### Training data

Not only the pretest and posttest data are of interest. Since the data was easily available, we explored our two main outcome variables, bias, and discrimination, over the course of the training. We employed a similar but still distinct memory task and used a 4-point instead of the 7-point confidence scale. This section presents the analyses for the two metacognitive training groups. Description and preliminary analyses of the training data can be found in the Supplementary Material 5. We used consistent main predictors across both analyses: measurement point** (**12 training sessions**)** at Level 1 and condition (MC Monitoring Feedback, MC Performance Feedback) at Level 2. We allowed both the intercept and the effect of measurement point to vary across participants. We defined the MC Performance Feedback group as reference group. To test our hypotheses, we conducted two sets of analyses using a cross-level interaction model between measurement point and group, one for bias and another for discrimination. We compared these models to more parsimonious models without the interaction terms. The analyses showed no significant improvement in model fit with the interaction term for bias, χ^2^(1) = 1.56, *p* = 0.21, suggesting that over the course of the 12 training sessions, there was no change in bias score. Consequently, for reasons of model parsimony, we decided to continue our analysis using a conditional model without the interaction terms using the following syntax: bias_score ~ measurement point + group + (measurement point |subject). Concerning discrimination, the analyses showed significant improvement in model fit with the interaction term, χ^2^(1) = 4.66, *p* = 0.03, suggesting a change in discrimination over the course of the 12 training sessions. Consequently, we decided to continue our analysis using the following syntax: discrimination ~ measurement point * group + (measurement point |subject). Complete details of all computed LMMs are provided in the Supplementary Material 6 and 7.

#### Absolute monitoring accuracy over the course of the training

The results for the conditional model for the absolute monitoring accuracy (bias) revealed that measurement point was not a significant predictor (γ = −0.00, SE = 0.00, *t*(144) = −0.15, *p* = 0.88), indicating that overall, the bias score did not change over the course of training. Moreover, no significant main effect for feedback was found (γ = 0.05, SE = 0.04, *t*(144) = 1.17, *p* = 0.24, suggesting that even though kindergarteners received extensive feedback, neither form of feedback influenced the children’s absolute monitoring accuracy during the 12 sessions.

#### Relative monitoring accuracy over the course of the training

**Fig. 2:**
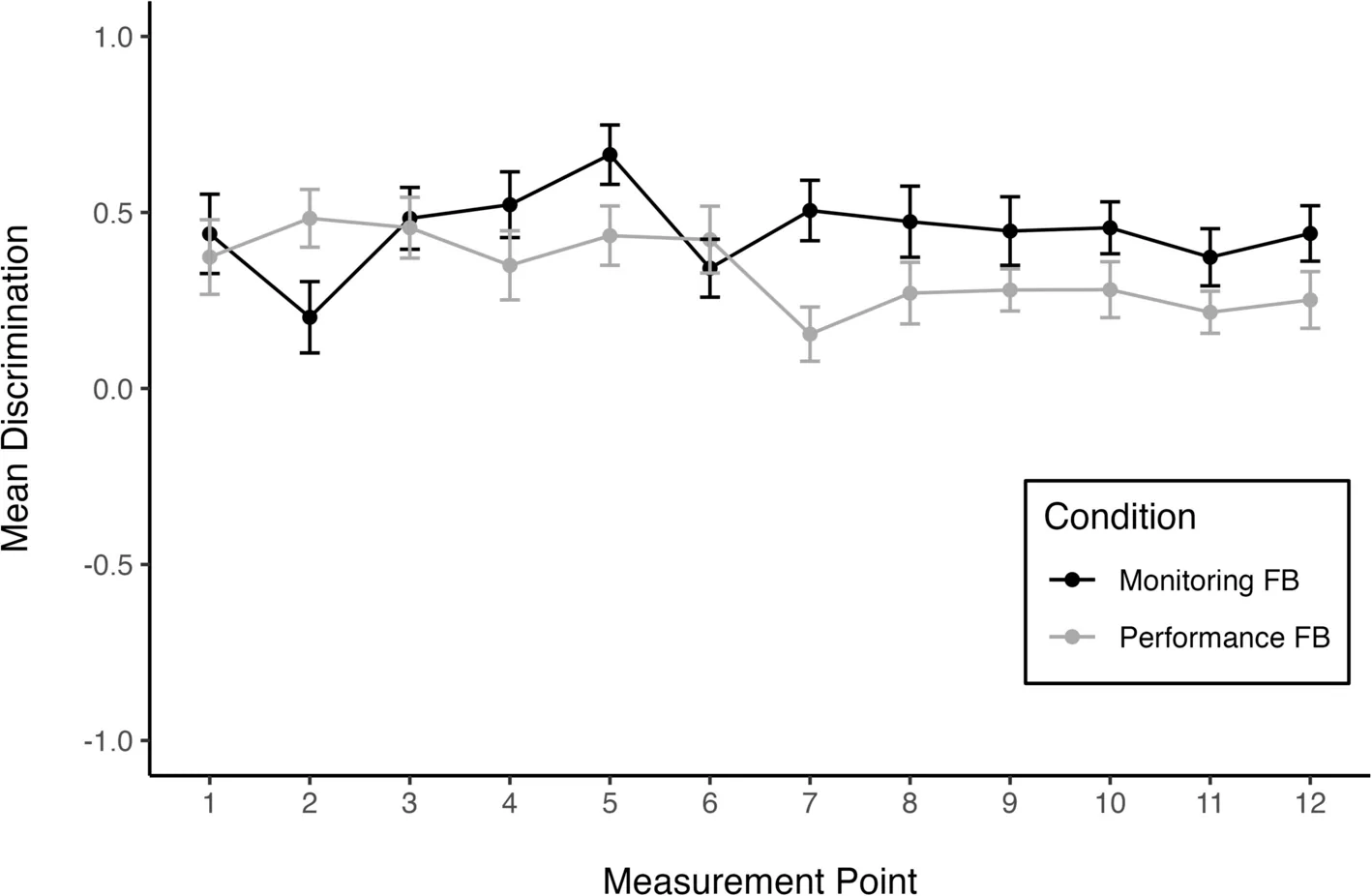
Means of discrimination as a function of training sessions and group. Note. Error bars indicate standard errors of the mean of discrimination

The cross-level interaction model yielded a significant interaction term (γ = 0.02, SE = 0.01, *t*(138) = 2.17, *p* = 0.03). This estimate indicates a significant difference between feedback groups. That is, children in the MC Monitoring Feedback group showed a marginal increase in discrimination per session compared to the children in the MC Performance Feedback group, whereas children in the MC Performance Feedback group showed a downward trend in their discrimination skills, indicating that discrimination suffers under performance feedback (see Fig. 2).

## Discussion

The primary goal of this study was to investigate whether and to what extent a learning app targeting metacognitive monitoring and providing feedback (performance feedback or monitoring feedback) is beneficial for improving kindergartners’ absolute (bias) and their emerging relative monitoring (discrimination) skills both during the training and when comparing pre- and posttest monitoring. Additionally, we explored whether the training might impact the role of retrieval fluency (i.e., choice latency) by strengthening this mnemonic cue to inform subsequent monitoring judgments (cue utilization). Although children remained motivated and positive throughout the study, worked independently with our learning app, and handled the technical aspects of the test and training sessions very well (indicating high feasibility), neither training form was found to be effective. This was true for both our primary outcome variables, monitoring bias and monitoring discrimination, as well as for cue utilization.

Before discussing the null effects of the training in more detail, we consider it important to mention that we replicated the well-established findings that kindergarteners (a) can metacognitively discriminate between correct and incorrect recognition (relative monitoring accuracy), giving higher CJs for correct than for incorrect answers (Destan et al., 2014). This is an important prerequisite as it underscores that our sample had the foundational skills for – theoretically- benefitting from the training. Moreover, and as has been shown repeatedly in the literature, (b) bias remained high, and children substantially overestimated their performance throughout (Lipko et al., 2009; Lipowski et al., 2013; Roebers, 2017). Apparently, children are very reluctant to report anything else but certainty, hinting once more at the possible evolutionary advantage of being confident (Bjorklund, 2023). Concerning cue utilization, our findings revealed that (c) kindergarteners indeed took choice latency as a valid cue into account when giving confidence judgments, at least to some extent. However, when directly comparing second graders’ Gamma correlations from Roebers et al. (2019) with the present study, unsurprisingly, cue utilization appeared relatively weak in our younger children. This suggests that while kindergarteners have already detected mnemonic cues, their use will likely undergo further developmental refinement. Nevertheless, it is important to interpret these findings with caution, as the relatively low correlation suggests that while cue utilization is a promising indicator of metacognitive development, it may require more time and experience as well as direct hints or explanations (“*You took a long time before responding and considered different response alternative; thus, you are most likely uncertain concerning this response!*”) to become more reliable and efficient in younger children. Although our study was not designed as an educational intervention, a potential application of these findings could involve providing children with repeated exposure to specific cues, informing them about the validity of specific cues, or even directly instructing them on how to use cues effectively. One promising avenue for future research could be the development of interactive apps or digital games specifically designed to train young children in identifying and utilizing specific cues. Such an app could offer adaptive, real-time feedback on cue use, reinforcing when children successfully recognize and use valid cues to adjust their confidence judgments.

Turning to the lack of training effects, we report that, contrary to our expectations, neither form of feedback improved the monitoring accuracy of the kindergarten children. This result was unexpected in light of previous studies, as three previous studies had suggested that monitoring feedback might improve young children’s monitoring (Buehler et al., 2025; Geurten & Meulemans, 2017; van Loon & Roebers, 2020). For instance, a short training study with older children in a memory learning paradigm found that monitoring feedback significantly enhanced monitoring accuracy (Buehler et al., 2025). However, it is important to note that these children were older and already in first grade, giving them much more exposure to structured learning tasks, where they likely had more experience with memory challenges and failures, teacher feedback, and formal performance situations. First graders’ monitoring skills are also further developed, possibly providing a more solid monitoring background on which the training can build. Together, this may have enhanced their understanding and application of feedback.

In fact, for kindergarten children, our feedback might have been too demanding, limiting its effectiveness. Six-year-olds may not yet have the cognitive capacity to fully benefit from metacognitive training. In line with this interpretation, van Loon and Roebers (2020) reported that monitoring feedback effectiveness interacted significantly with kindergarten children’s working memory capacity, with those children benefitting more from the feedback who had larger working memory capacity than age-mates with lower capacity. This suggests that feedback integration is demanding and can interfere with other simultaneous information processing (monitoring). For example, performance feedback that remains visible on the screen while children monitor has been reported to work better, even in young children, underscoring the need for reducing working memory demands while training children to monitor accurately (Muis et al., 2015; van Loon & Roebers, 2017). This interpretation of working memory demanding feedback aligns well with the findings of Anquillare and Selmeczy (2023), who report that simple memory accuracy feedback allows children to focus more effectively on task demands compared to point-based feedback, which can be more challenging to process. In their study, feedback was categorized into three types: point-based (focusing on points accumulation), memory accuracy feedback (indicating if responses were correct or incorrect), and a no-feedback control to gauge baseline memory performance. They found that feedback requiring strategic engagement, like point-based feedback, is harder for children to process. This difficulty appeared to stem from the need to simultaneously manage task performance, integrate complex feedback, and engage in monitoring, possibly overloading their cognitive capacities (Fyfe et al., 2015).

Another explanation for the lack of an overall intervention effect may be that young children do not view feedback as relevant to their performance. Kindergarten-aged children may prioritize their efforts over past performance, leading to wishful thinking and focusing on effort alone for success (Schneider, 1998). Negative feedback may be perceived as self-threatening, leading to superficial processing compared to positive feedback (Stipek & Tannatt, 1984; Stipek et al., 1984). It thus appears that they may need additional assistance, direction, and monitoring strategies (including hints to valid cues, see above) to benefit from feedback (Labuhn et al., 2010; Pennequin et al., 2010; Van der Kleij et al., 2015).

Another significant factor, as Efklides (2008) discusses, could be the social aspect of feedback in self-regulated learning. Computer-based feedback may not be as effective as personal, face-to-face feedback from a teacher, parent, or research assistant who can ensure understanding, provide motivation, offer tailored explanations, point to cue utilization promptly, and reinforce accurate monitoring specifically and instantly (Bloom, 1984; Siraj & Asani, 2014; van Loon & Roebers, 2020). This is particularly relevant when considering the opposing findings of van Loon and Roebers (2020), where tasks were conducted in a one-on-one setting with individualized and personal feedback provided by the experimenter.

Lastly, drawing from the extensive literature on executive function interventions, longer durations of training might lead to better effects in monitoring accuracy (Diamond & Ling, 2016, 2020). Extending the training duration (by giving more trials per session and more sessions overall) could enhance the effectiveness of the training. Additionally, increasing the *intensity* of the training (by including more extreme differences in item difficulty and/or by applying a bonus-to-penalty ratio for correct monitoring) might further support improvements in monitoring accuracy.

Because the development of learning apps for kindergarten children is still in its early stages, a discussion on the feasibility seems also warranted. Generally, children worked well with the learning app, and technical issues rarely arose. However, a close inspection of how children worked through our tasks (see Table 2 and Fig. 2) revealed that especially children who received one of the training forms became significantly faster at selecting a response in the posttest than the active control group. In other words, children did not benefit from either form of feedback in terms of taking their time to consider their certainty thoroughly. It appears that the computer-based feedback was not powerful enough to motivate them to closely monitor, but rather, unfortunately, they learned how to complete the task “efficiently.” App developers should take this into account. Educators may also play a crucial role in supporting children’s metacognitive development by incorporating social interactions and active guidance throughout the learning process. Ideally, this should be flanked by computer-based training (Labuhn et al., 2010; Pennequin et al., 2010). Teachers can enhance learning by engaging children in dynamic, reflective dialogues, prompting them to explain their reasoning, encouraging them to verbalize their confidence levels, and leading them to recognize valid cues for their monitoring. Additionally, teachers should provide responsive, adaptive feedback that helps children recognize and correct misconceptions, reinforcing self-monitoring strategies, and helping them to disregard invalid cues. Structuring learning activities in a way that includes peer discussions, teacher-led questioning, and explicit feedback cycles can further support retention, a deeper understanding of new content, and a more accurate monitoring of their performance (Hirsh-Pasek et al., 2015; Langenhoff et al., 2024; Pozuelos et al., 2019). A promising line of future research might address the effects of a multi-modal intervention, combining teachers’ feedback, instructions about valid cues, and learning app to apply newly learned monitoring skills.

Recognizing the value of replication in research, we encourage other research groups to replicate our study to further validate and extend our findings. Looking ahead, future research could, on the one hand, focus on developing and implementing adaptive learning apps that feature more personalized, differentiated feedback systems to enhance metacognitive skills such as monitoring and confidence. In fact, tailoring task difficulty to each child’s current metacognitive and cognitive abilities by incorporating adaptive features that adjust task complexity based on individual performance might also be a promising avenue for future work. Educators, on the other hand, play a crucial role in integrating these apps into classrooms by aligning tasks with the curriculum, integrating metacognitive experiences in different modalities in every day kindergarten activities, and ensuring that feedback is developmentally appropriate to support students’ learning progress and in parallel to app-based feedback to maximize children’s benefit.

Surprisingly, we found one training effect, but only in the monitoring feedback group, on children’s choice latencies in the recognition test as a function of their level of certainty. The inverted U-shaped curve is typically only observed in older participants (van Loon et al., 2013) and interpreted as showing that an individual has developed a monitoring skill that enables fast awareness of the two extremes on the certainty-uncertainty continuum. It might constitute a precursor of more fine-tuned and accurate uncertainty monitoring, and possibly, with the present monitoring training, we might have initiated such developmental progress. This is highly speculative, though, and needs further investigation.

### Strength and limitations

This study provides new and important insights into challenges when investigating the effectiveness of intensive computerized metacognitive monitoring training aiming to improve kindergarteners’ monitoring accuracy. The strengths of our study lie in its innovative approach of integrating many and various metacognitive monitoring experiences and detailed feedback into a child-friendly and entertaining computer-based learning app that may be easily implemented. Learning apps are resource-sparing, and although not effective in their present form, our approach provided valuable insights into kindergarten children’s ability to learn from metacognitive experiences and how to design learning apps for young children’s benefit. Thereby, choice latencies and their potential as valid cue for informing monitoring might be a promising avenue for future research in this important domain.

Despite these contributions, certain limitations should be acknowledged. One key consideration is the duration of training required to effectively enhance monitoring abilities. The optimal length of training remains a subject of debate, with evidence suggesting that long-term interventions—spanning several months or integrated into school curricula—can lead to substantial improvements (Diamond & Lee, 2011; Diamond & Ling, 2020; Hacker et al., 2000; Nietfeld et al., 2006). In comparison, the duration of our training was relatively short, which may have influenced the extent of the observed effects.

Another limitation concerns the nature of the task in which monitoring exercises were embedded in both the pre-posttest task and training. These tasks were memory tasks, which, although very relevant, do not fully capture the modalities of real-world cognitive processing and are relatively challenging for kindergarteners. Future studies should broaden the scope of tasks, including, for example, perceptual tasks (Lyons & Ghetti, 2011), working memory-related tasks (Applin & Kibbe, 2021), or tasks assessing foundational skills in reading and mathematics (Näslund & Schneider, 1996). Expanding the task range would provide a more comprehensive understanding of individual differences in monitoring ability and the role of task complexity for which performance is monitored and help disentangle monitoring accuracy from general task performance.

Lastly, although data collection took place in familiar environments, such as children’s kindergartens, it is important to recognize that the studies were conducted under controlled experimental conditions. To improve the ecological validity of findings, future research should integrate metacognitive experiences more seamlessly into natural classroom settings, opt for multi-modal training opportunities, and evaluate their long-term effectiveness.

## Appendix A

Table 3

**Table 3:** Detailed description of feedback for both experimental feedback groups

Group	RC	CJ	Verbal Feedback
Performance Feedback			
	1	1–4	You have chosen the right food
	0	1–4	You have chosen the wrong food. Don’t worry. It wasn’t easy
Monitoring Feedback (match between performance and CJs)			
	1	4	You have chosen the right food. Very good that you are very certain
	1	3	You have chosen the right food. Good that you are certain
	0	1	You have chosen the wrong food. Very good that you are very uncertain
	0	2	You have chosen the wrong food. Good that you are uncertain
Monitoring Feedback (no match performance and CJs)			
	1	1	You have chosen the right food. It is a pity that you are very uncertain
	1	2	You have chosen the right food. It is a pity that you are uncertain
	0	4	You have chosen the wrong food. Don’t worry. But it is a pity that you are very certain
	0	3	You have chosen the wrong food. Don’t worry. But it is a pity that you are certain

*RC* recognition correctness, *CJs* confidence judgements: 1 = very uncertain, 2 = uncertain, 3 = certain, 4 = very certain

## Appendix B

Figure 3

**Fig. 3:**
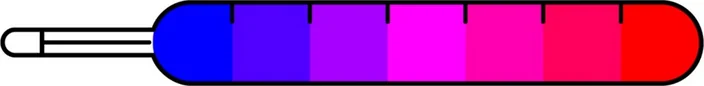
Thermometer scale used in pretest and posttest

Figure 4

**Fig. 4:**
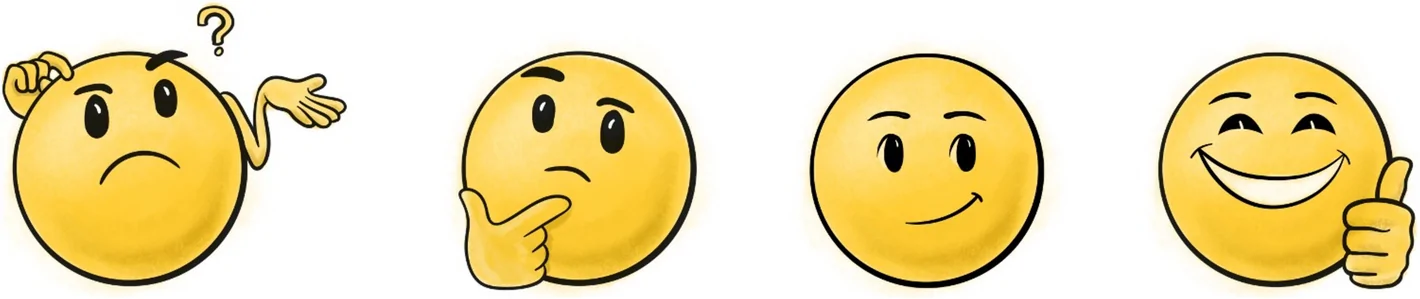
Smiley scale used in metacognitive monitoring training

## Appendix C

Figure 5

**Fig. 5:**
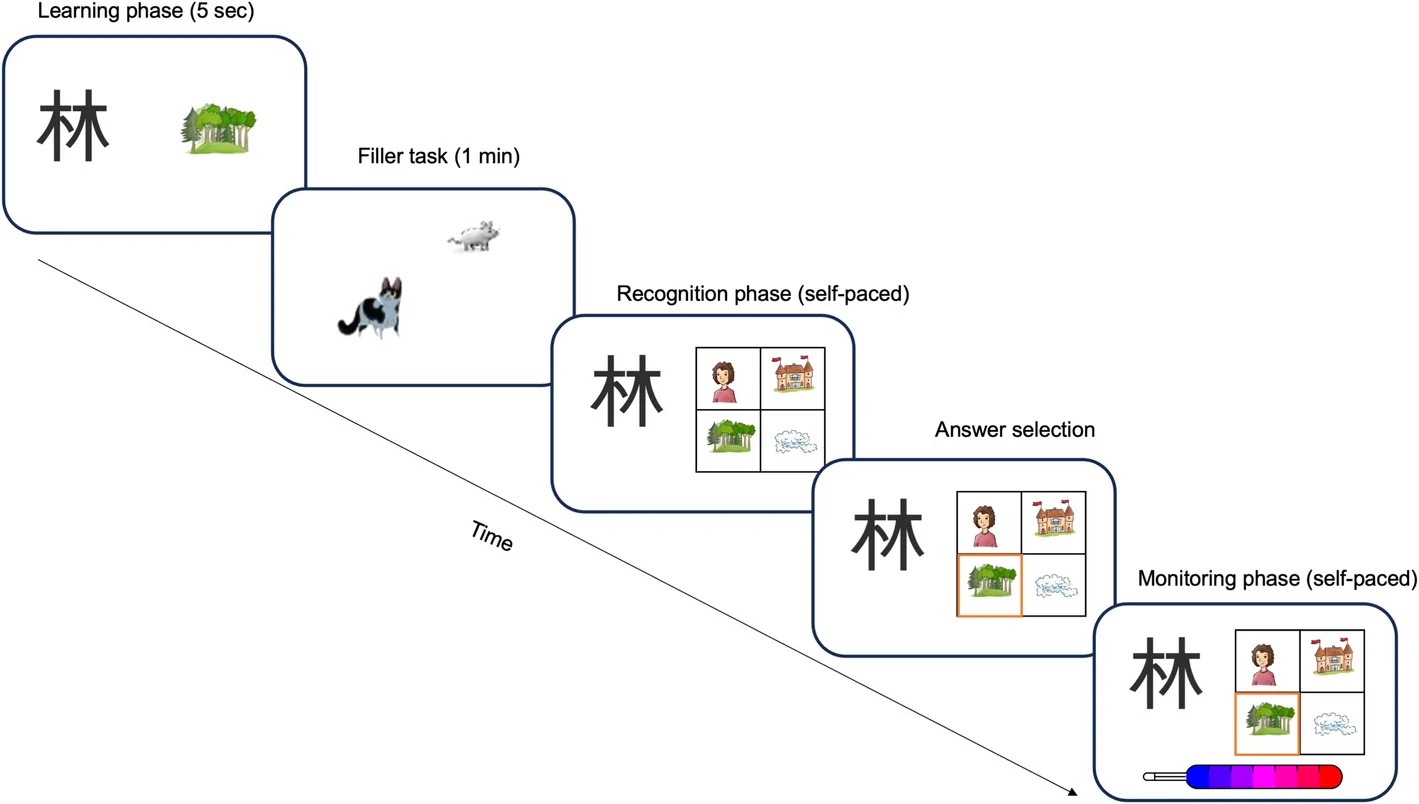
Task procedure at pretest and posttest

## Appendix D

Figure 6

**Fig. 6:**
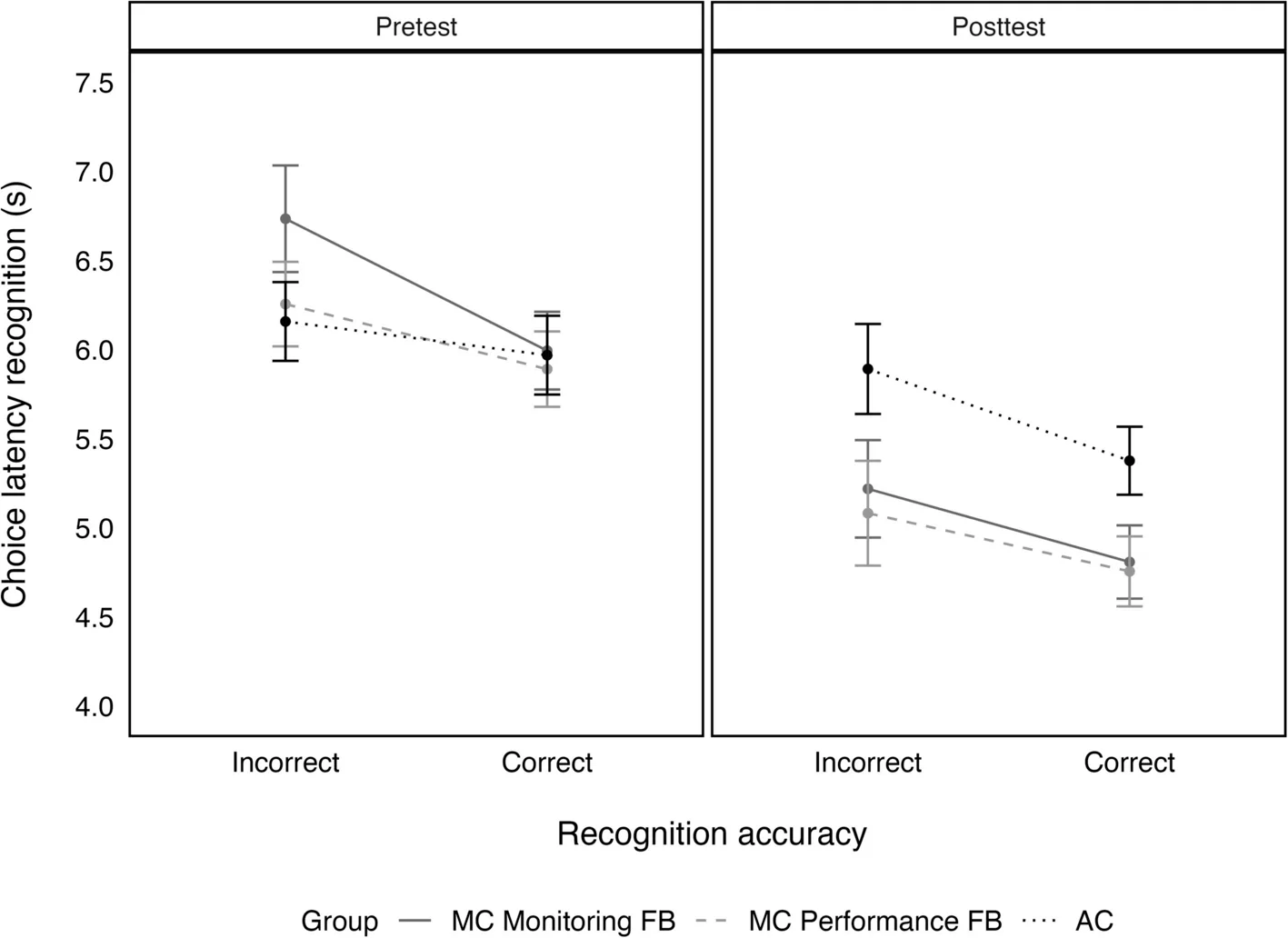
Choice latencies for correct and incorrect responses as a function of group and measurement point*.* Note*.* Error bars indicate standard errors

## Supplementary Information

Below is the link to the electronic supplementary material.

Supplementary file1 (PDF 50.9 KB )

## Data Availability

https://osf.io/3647m/?view_only=e209d9a33bba4a5e9d3fc3338c730bd9.
