# Supplementary material for: The role of metacognitive experience: Can feedback training affect kindergarteners'monitoring accuracy?
Source: Metacogn Learn. 2025 Jun 5;20(1):20. doi: 10.1007/s11409-025-09422-4 (PMC12141379; doi:10.1007/s11409-025-09422-4)
Supplement: Supplementary file 1 — Supplementary file1 (PDF 50.9 KB ) [file 11409_2025_9422_MOESM1_ESM.docx]

# Supplemental Material

**Supplemental Material Table 1**

*Model Parameter and Goodness of Fit for Linear Mixed Models for Absolute Monitoring Accuracy (Bias)*

| Effect (parameter) | Model 1 | Model 2 | Model 3 | Model 4 |
| --- | --- | --- | --- | --- |
|  | Fixed effects |  |  |  |
| Intercept | 0.31***(0.02) | 0.34***(0.03) | 0.35***(0.03) | 0.35***(0.03) |
| Measurement point |  | -0.06*(0.03) | -0.06*(0.03) | -0.06*(0.03) |
| MT monitoring feedback |  |  | -0.01 (0.04) | -0.03 (0.04) |
| MT performance feedback |  |  | -0.05 (0.04) | 0.00 (0.04) |
| Measurement point x MT monitoring feedback |  |  |  | -0.02 (0.05) |
| Measurement point x MT performance feedback |  |  |  | 0.02 (0.06) |
|  | Random effects |  |  |  |
| Variance components |  |  |  |  |
| Residual variance | 0.07 | 0.07 | 0.07 | 0.07 |
| Intercept variance | 0.03 | 0.03 | 0.03 | 0.03 |
| ICC | 0.27 | 0.28 | 0.28 | 0.28 |
| Marginal R^2^/Conditional R^2^ | 0.00/0.27 | 0.01/0.28 | 0.01/0.29 | 0.01/0.29 |
| Observation | 428 | 428 | 428 | 428 |
|  | Goodness of fit |  |  |  |
| AIC | 206.00 | 203.30 | 205.68 | 209.43 |
| BIC | 218.18 | 219.60 | 230.03 | 241.90 |
| Deviance | 200.0 | 195.32* | 193.68 | 193.43 |

*Note*. Standard errors in parentheses. All *p* values in this table are two-tailed. Model 1= intercept-only model, Model 2 = unconditional model, Model 3 = conditional model, Model 4 = cross-level interaction model.

**p* < .05. ****p* < .001

**Supplemental Material Table 2**

*Model Parameter and Goodness of Fit for Linear Mixed Models Relative Monitoring Accuracy (Discrimination)*

| Effect (parameter) | Model 1 | Model 2 | Model 3 | Model 4 |
| --- | --- | --- | --- | --- |
|  | Fixed effects |  |  |  |
| Intercept | 0.52***(0.06) | 0.51***(0.08) | 0.52***(0.03) | 0.52***(0.03) |
| Measurement point |  | 0.02 (0.10) | 0.02 (0.10) | 0.10 (0.10) |
| MT monitoring feedback |  |  | 0.03 (0.15) | 0.08 (0.15) |
| MT performance feedback |  |  | 0.03 (0.15) | -0.14 (0.15) |
| Measurement point x MT monitoring feedback |  |  |  | 0.10 |
| Measurement point x MT performance feedback |  |  |  | -0.34 |
|  | Random effects | |  |  |
| Variance components |  |  |  |  |
| Residual variance | 1.16 | 1.16 | 1.16 | 1.15 |
| Intercept variance | 0.23 | 0.23 | 0.23 | 0.24 |
| ICC | 0.17 | 0.17 | 0.17 | 0.18 |
| Marginal R^2^/Conditional R^2^ | 0.00/0.17 | 0.00/0.17 | 0.00/0.17 | 0.00/0.18 |
| Observation | 428 | 428 | 428 | 428 |
|  | Goodness of fit | |  |  |
| AIC | 1,357.90 | 1,359.90 | 1,363.80 | 1,364.60 |
| BIC | 1,370.10 | 1,376.10 | 1,388.20 | 1,397.10 |
| Deviance | 1,351.90 | 1,351.90 | 1,351.80 | 1,348.60 |

*Note*. Standard errors in parentheses. All *p* values in this table are two-tailed. Model 1= intercept-only model, Model 2 = unconditional model, Model 3 = conditional model, Model 4 = cross-level interaction model.

****p* < .001

**Supplemental Material Table 3**

*GLMM Parameter and Goodness of Fit for Cue Validity*

| Effect (parameter) | Model 1  OR [CI] | Model 2  OR [CI] | Model 3  OR [CI] | Model 4  OR [CI] |
| --- | --- | --- | --- | --- |
|  | Fixed effects |  |  |  |
| Intercept | 0.58***[0.54, 0.63] | 0.63***[0.53, 0.73] | 0.61***[0.50, 0.74] | 0.64**[0.46, 0.88] |
| Choice latency [sec] |  | 0.97**[0.96, 0.99] | 0.97**[0.95, 0.99] | 0.96 [0.92, 1.01] |
| Measurement point |  | 1.21***[1.09, 1.34] | 1.21***[1.05, 1.36] | 1.20 [0.79, 1.84] |
| MT monitoring feedback |  |  | 1.08 [0.95,1.32] | 1.05 [0.67,1.65] |
| MT performance feedback |  |  | 1.02 [0.83, 1.25] | 0.99 [0.62, 1.57] |
| Measurement point x MT monitoring feedback |  |  |  | 0.99 [0.93, 1.06] |
| Measurement point x MT performance feedback |  |  |  | 0.98 [0.55, 1.76] |
| Choice latency x MT monitoring feedback |  |  |  | 1.00 [0.94, 1.06] |
| Choice latency x MT performance feedback |  |  |  | 1.00 [0.94, 1.07] |
| Measurement point x choice latency x MT monitoring feedback |  |  |  | 1.01 [0.93, 1.11] |
| Measurement point x choice latency x MT performance feedback |  |  |  | 1.03 [0.94, 1.13] |
|  | Random effects |  |  |  |
| Variance components |  |  |  |  |
| Residual variance | 3.29 | 3.29 | 3.29 | 3.29 |
| Subject level variance | 0.20 | 0.29 | 0.28 | 0.29 |
| Slope variance |  | 0.30 | 0.30 | 0.30 |
| Covariance |  | -0.46 | -0.46 | -0.48 |
| ICC | 0.06 |  |  |  |
| Marginal R^2^/Conditional R^2^ |  | 0.01/0.07 | 0.01/0.9 | 0.01/0.9 |
| Observation | 6,658 | 6,658 | 6,658 | 6,658 |
|  | Goodness of fit |  |  |  |
| AIC | 8,696.90 | 8,657.80 | 8,661.20 | 8,673.80 |
| BIC | 8,710.50 | 8,698.70 | 8,715.60 | 8,778.80 |
| Deviance | 8,692.90 | 8,645.80*** | 8,645.20 | 8,643.80 |

*Note*. OR = Odds ratio, CI = confidence interval. Model 1= intercept-only model, Model 2 = unconditional model, Model 3 = conditional model, Model 4 = cross-level interaction model.

***p* < .01. ****p* < .001

**Supplemental Material Table 4**

*Model Parameter and Goodness of Fit for Linear Mixed Models Cue Utilization*

| Effect (parameter) | Model 1 | Model 2 | Model 3 | Model 4 |
| --- | --- | --- | --- | --- |
|  | Fixed effects |  |  |  |
| Intercept | 5.10***(0.09) | 5.30***(0.11) | 5.41***(0.17) | 5.42***(0.23) |
| Measurement point |  | -0.15 (0.13) | -0.15 (0.13) | 0.02 (0.18) |
| Choice latency for recognition [sec] |  | -0.06***(0.01) | -0.06***(0.01) | -0.07** (0.02) |
| Recognition accuracy |  | 0.55***(0.05) | 0.55***(0.05) | 0.54***(0.05) |
| MT monitoring feedback |  |  | 0.02 (0.22) | 0.32 (0.34) |
| MT performance feedback |  |  | -0.28 (0.22) | -0.35 (0.33) |
| Measurement point x MT monitoring feedback |  |  |  | -0.64*(0.25) |
| Measurement point x MT performance feedback |  |  |  | -0.13 (0.26) |
| Measurement point x choice latency |  |  |  | -0.01(0.03) |
| Choice latency x MT monitoring feedback |  |  |  | -0.04 (0.04) |
| Choice latency x MT performance feedback |  |  |  | 0.03 (0.04) |
| Measurement point x choice latency x MT monitoring feedback |  |  |  | 0.11**(0.04) |
| Measurement point x choice latency x MT performance feedback |  |  |  | -0.01 (0.04) |
|  | Random effects | |  |  |
| Variance components |  |  |  |  |
| Residual variance | 3.99 | 3.01 | 3.01 | 3.72 |
| Subject level variance | 1.69 | 2.42 | 2.41 | 2.51 |
| Slope variance |  | 3.44 | 3.44 | 0.02 |
| Covariance |  | -0.56 | -0.56 | -0.61 |
| ICC |  |  |  |  |
| Marginal R^2^/Conditional R^2^ | 0.00/0.30 | 0.02/0.47 | 0.02/0.47 | 0.03/0.35 |
| Observation | 6,658 | 6,658 | 6,658 | 6,658 |
|  | Goodness of fit | |  |  |
| AIC | 28,683 | 27,353 | 27,355 | 28,421 |
| BIC | 28,704 | 27,408 | 27,423 | 28,537 |
| Deviance | 28,677 | 27,337*** | 27,335 | 28,387 |

*Note*. Standard errors in parentheses. All *p* values in this table are two-tailed. Model 1= intercept-only model, Model 2 = unconditional model, Model 3 = conditional model, Model 4 = cross-level interaction model.

**p* = .05. ***p* < .01. ****p* < .001.

**Supplemental Material 5: Preparation and Preliminary Analysis for Training Data**

One hundred forty-four children participated in the metacognitive training, with *n* = 72 receiving monitoring feedback and *n* = 72 receiving performance feedback. In preparing the data for analysis in the training task, choice latencies below 500 ms were removed (18 of 18,860 observations, 0.01%), and outliers, defined as choice latencies > ±3 SD from the individual's mean (40 of 18,842 observations, 0.21%) and group level mean (302 of 18,802 observations, 1.61%), were also excluded. We determined the order of training topics (animal habitats) using a Latin square. Analysis across the 12 topics revealed that children's memory performance ranged from 43% to 69%. Subsequent individual t-tests indicated significant differences in performance between groups at sessions five and 12 (Supplemental Table S5). MC Performance Feedback condition was set as the reference group

**Supplemental Material Table 5**

*Summary of t-Tests Results for Performance as a Function of Session*

| Session | Performance % Monitoring FB | Performance % Performance FB | Mean difference | *t* | df | *p* |
| --- | --- | --- | --- | --- | --- | --- |
| 1 | 0.55 (0.46) | 0.60 (0.47) | -0.05 | -1.28 | 126 | 0.20 |
| 2 | 0.43 (0.48) | 0.45 (0.48) | -0.03 | -0.87 | 140 | 0.39 |
| 3 | 0.56 (0.46) | 0.58 (0.46) | -0.01 | -0.34 | 132 | 0.73 |
| 4 | 0.55 (0.47) | 0.59 (0.46) | -0.04 | -0.93 | 128 | 0.36 |
| 5 | 0.54 (0.48) | 0.60 (0.47) | -0.06 | -2.03 | 135 | **0.04** |
| 6 | 0.59 (0.45) | 0.60 (0.45) | -0.01 | -0.17 | 134 | 0.87 |
| 7 | 0.55 (0.47) | 0.60 (0.44) | -0.05 | -1.43 | 132 | 0.16 |
| 8 | 0.57 (0.46) | 0.57 (0.46) | 0.00 | -0.09 | 127 | 0.93 |
| 9 | 0.55 (0.46) | 0.56 (0.47) | -0.02 | -0.42 | 133 | 0.67 |
| 10 | 0.57 (0.46) | 0.60 (0.45) | -0.03 | -0.86 | 129 | 0.39 |
| 11 | 0.57 (0.46) | 0.58 (0.47) | -0.01 | -0.40 | 130 | 0.69 |
| 12 | 0.50 (0.47) | 0.60 (0.47) | -0.10 | -2.96 | 129 | **0.01** |

*Note:* Bold = significant sessions.

**Supplemental Material Table 6**

*Model Parameter and Goodness of Fit for Linear Mixed Models for Absolute Monitoring Accuracy (Bias) in Training*

| Effect (parameter) | Model 1 | Model 2 | Model 3 | Model 4 |
| --- | --- | --- | --- | --- |
|  | Fixed effects |  |  |  |
| Intercept | 0.16***(0.02) | 0.16***(0.03) | 0.14***(0.03) | 0.15***(0.03) |
| Measurement point |  | -0.00 (0.01) | -0.00 (0.01) | -0.00 (0.01) |
| MT monitoring feedback |  |  | 0.04 (0.04) | 0.03 (0.04) |
| Measurement point x MT monitoring feedback |  |  |  | 0.01 (0.01) |
|  | Random effects | |  |  |
| Variance components |  |  |  |  |
| Residual variance | 0.04 | 0.03 | 0.03 | 0.03 |
| Intercept variance | 0.05 | 0.05 | 0.05 | 0.05 |
| Slope variance |  | 0.00 | 0.00 | 0.00 |
| Covariance |  | -0.16 | -0.18 | -0.17 |
| ICC | 0.59 | 0.66 | 0.65 | 0.65 |
| Marginal R^2^/Conditional R^2^ | 0.00/0.59 | 0.00/0.66 | 0.01/0.66 | 0.01/0.66 |
| Observation | 144 | 144 | 144 | 144 |
|  | Goodness of fit | |  |  |
| AIC | -299.30 | -409.35 | -408.70 | -408.26 |
| BIC | -283.14 | -377.03 | -370.99 | -365.16 |
| Deviance | -305.30 | -421.25*** | -422.70 | -424.26 |

*Note*. Standard errors in parentheses. All *p* values in this table are two-tailed. Model 1= intercept-only model, Model 2 = unconditional model, Model 3 = conditional model, Model 4 = cross-level interaction model.

****p* < .001.

**Supplemental Material Table 7**

*Model Parameter and Goodness of Fit for Linear Mixed Models for Relative Monitoring Accuracy (Discrimination) in Training*

| Effect (parameter) | Model 1 | Model 2 | Model 3 | Model 4 |
| --- | --- | --- | --- | --- |
|  | Fixed effects |  |  |  |
| Intercept | 0.39*** (0.03) | 0.44***(0.03) | 0.39*** (0.05) | 0.45*** (0.06) |
| Measurement point |  | -0.01 (0.01) | -0.01 (0.01) | -0.02** (0.01) |
| MT monitoring feedback |  |  | 0.11 (0.06) | -0.02 (0.08) |
| Measurement point x MT monitoring feedback |  |  |  | 0.02* (0.01) |
|  | Random effects | |  |  |
| Variance components |  |  |  |  |
| Residual variance | 0.45 | 0.43 | 0.43 | 0.43 |
| Intercept variance | 0.07 | 0.11 | 0.12 | 0.11 |
| Slope variance |  | 0.00 | 0.00 | 0.00 |
| Covariance |  | -0.56 | -0.61 | -0.59 |
| ICC | 0.14 | 0.14 | 0.17 | 0.17 |
| Marginal R^2^/Conditional R^2^ | 0.00/0.14 | 0.00/0.18 | 0.01/0.18 | 0.01/0.66 |
| Observation | 144 | 144 | 144 | 144 |
|  | Goodness of fit | |  |  |
| AIC | 3,432.80 | 3,426.30 | 3,426.38 | 3,422.10 |
| BIC | 3,448.90 | 3,458.70 | 3,462.50 | 3,65.20 |
| Deviance | 3,426.80 | 3,414.30** | 3,410.80* | 3,406.10* |

*Note*. Standard errors in parentheses. All *p* values in this table are two-tailed. Model 1= intercept-only model, Model 2 = unconditional model, Model 3 = conditional model, Model 4 = cross-level interaction model.

**p* = .05. ***p* = .01. ****p* < .001.
